# Simple Derivatization–Gas Chromatography–Mass Spectrometry for Fatty Acids Profiling in Soil Dissolved Organic Matter

**DOI:** 10.3390/molecules25225278

**Published:** 2020-11-12

**Authors:** Neil Yohan Musadji, Claude Geffroy-Rodier

**Affiliations:** 1 Institut de Chimie des Milieux et Matériaux de Poitiers (IC2MP), Université de Poitiers, UMR CNRS 7285, Equipe EBiCOM, 4 rue Michel Brunet, 86076 Poitiers, France; nyohan1@gmail.com; 2 Institut National Supérieur d’Agronomie et de Biotechnologies (INSAB), Université des Sciences et Techniques de Masuku (USTM), 941 Franceville, Gabon

**Keywords:** soil dissolved organic matter, fatty acids, methylation

## Abstract

Dissolved organic matter is an important component of the global carbon cycle that allows the distribution of carbon and nutrients. Therefore, analysis of soil dissolved organic matter helps us to better understand climate change impacts as it is the most dynamic and reactive fraction in terrestrial ecosystems. Its characterization at the molecular level is still challenging due to complex mixtures of hundreds of compounds at low concentration levels in percolating water. This work presents simple methods, such as thermochemolysis– or derivatization–gas chromatography, as an alternative for the analysis of fatty acids in dissolved organic matter without any purification step. The variables of the protocols were examined to optimize the processing conditions for the C_9_–C_18_ range. As a proof of concept, fatty acid distributions of soil percolating water samples from a long-term field experiment were successfully assessed. The variability of dissolved organic acid distributions was pronounced through depth profile and soil treatment but no major change in composition was observed. However, although the optimization was done from C_9_ to C_18_, detection within the C_6_-C_32_ fatty acids range was performed for all samples.

## 1. Introduction

Soil dissolved organic matter (SDOM, OM dissolved in soil solution which passes a 0.45 μm filter), which represents a small portion of soil organic matter (<2%), is the most dynamic and reactive fraction in terrestrial ecosystems [1,2]. Its characterization is an important issue for soil environmental studies as DOM is a useful indicator for SOM quality [1,3,4], pollution [5] and soil management impact [1,6,7,8]. Although quantifying SDOM amount (commonly by measuring dissolved organic carbon concentration, DOC) is necessary, it is also important to characterize its composition at the molecular level, as the components drive its reactivity and properties [9].

From an analytical perspective, SDOM characterization at the molecular level poses a special challenge. The complex and diverse soil dissolved organic matter requires a multi-method analytical characterization [10]. A single sample can be composed of tens of thousands of individual molecules that together rarely exceed 1 mg C/L. Actual molecular-level structural characterization, which requires detailed extraction and separation protocols, is limited to a few compounds or compound classes, and thus a very small portion of the total DOM pool [11]. Recently, studies have focused on targeted molecules relevant for particular biogeochemical processes but, as far as we know, none were dedicated to biomarkers analysis. Biomarkers preserving molecular fingerprints are indicative of past vegetation cover, soil organic matter input and microbial diversity. Their identification would be of major interest to an investigation of soil functioning. Among biomarkers, carboxylic acids, which represent, for the low molecular weight ones, a significant part of the water-soluble fractions of organic molecules released in the rhizosphere [12], exhibit important roles in soil nutrient availability, ecology and productivity. Generally, they are weak acids ranging from 0.46 to 1 million Dalton (Da). Although numerous techniques are devoted to volatile fatty acids (gas, liquid or ion chromatography, titration, mid-infrared spectroscopy) in aqueous samples, none or only few are used for soil leachates characterization [13,14,15,16,17] and are useful for fast total fatty acids monitoring. Most of the above methods either require sophisticated instruments, or are time-consuming or not useful for higher molecular weight fatty acids, making them unsuitable for fatty acids fingerprints. Therefore, the optimization of in situ characterization of the latter makes it possible to eliminate any pretreatment of aqueous samples.

To study DOM dynamics, numerous laboratory experiments using disturbed soil samples extracted with aqueous solutions have been conducted, but few have been performed on samples collected in field experiments [18,19]. Experiments at field scales are now needed to characterize undisturbed samples [20,21]. However, field experiments mean time consuming experiments with many samples to handle to take into account time and scale soil variabilities. Developing a rapid screening of fatty acids in SDOM from field experiments will allow the selection of representative samples for further investigations.

The main objectives of this study were (i) to develop a simple, fast and accurate strategy to detect fatty acids in water solutions with few pretreatments to preserve the native molecular structures and (ii) to validate it on percolating soil solutions from a long-term experiment performed on an amended soil and its reference [21,22]. Two analytical strategies have been evaluated in this study for the identification of fatty acids in SDOM. Thermochemolysis, with the alkaline reagent tetramethylammonium hydroxide (TMAH) which is efficient in hydrolyzing and derivatizing all fatty acids in soil humic substances, would allow the detection of total fatty acids (both free and covalently bound acids) [21,22]. Derivatization, commonly used for extracted organic solvent fatty acids, will allow to detect SDOM free fatty acids [11,23,24].

## 2. Results and Discussion

### 2.1. Analysis of SDOM Fatty Acids from Evaporated to Dryness Samples

#### 2.1.1. Thermochemolysis: Fingerprints of Total Fatty Acids in SDOM

##### Thermochemolysis in the C_9_–C_18_ Fatty Acids Range

**Figure 1:**
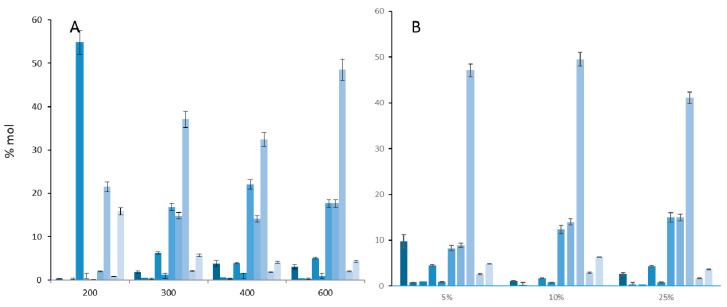
%mol (Ai/∑Ai) of fatty acid methyl esters (C_9_ to C_18_) in function of (**A**) the temperature of thermochemolysis (with 25% TMAH in MeOH) and (**B**) the percentage of TMAH in methanol (at 600 °C, 15 µL TMAH solution).

To have an overview of the SDOM fatty acids, optimal analytical conditions of thermochemolysis in the presence of TMAH were first determined on a standard mixture of C_9_ to C_18_ fatty acids. An amount of 25%TMAH in methanol at 600 °C was found to be the best conditions as it gave the highest peak intensity and a good overall molecular response (Figure 1).

##### SDOM Analysis by Thermochemolysis

The fatty acid distribution of percolating waters from urban green waste amended soil and its non-amended reference was investigated to validate thermochemolysis for SDOM analysis. In total, 500 mL of each sample (ranging from 8 to 61 mgC/L, Figure S1) was evaporated at 40 °C to dryness and optimal thermochemolysis was performed on the resulting residues.

**Figure 2:**
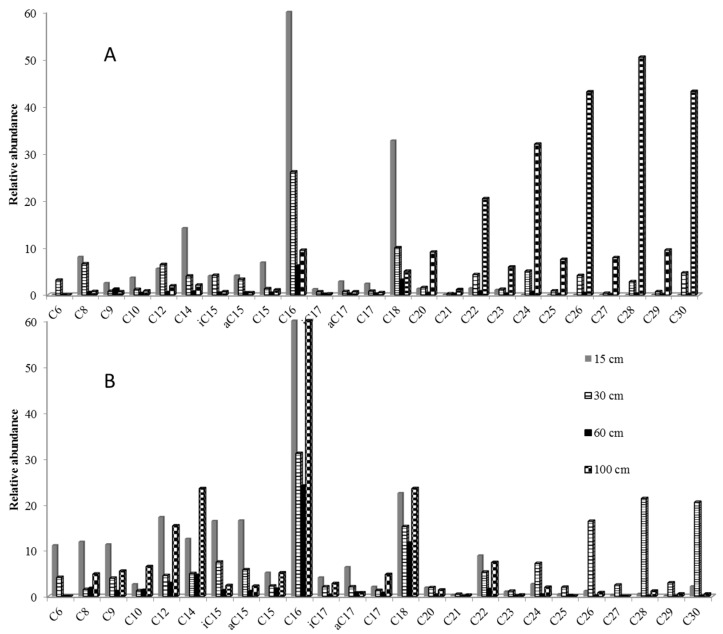
Thermochemolysis of 500 mL reference (**A**) and amended (**B**) soil solutions from 15 to 100 cm depth after a 40 °C evaporation step (TMAH 25%, 600 °C).

Fatty acids, observed as methyl esters, ranged in all samples from C_6_ to C_30_. The bimodal distribution was dominated by C_16_ for short mode (C_6_–C_18_) and C_28_ and C_30_ for long-chain acids (Figure 2). In reference soil solutions, even-over-odd predominance in the C_24_–C_32_ range was a marker of higher plant vegetation (Figure 2A). In amended soil solutions, branched isomers of C_15_ and C_17_ were as abundant as long-chain compounds in the first 30 cm depth, showing higher microbial activity than in reference soil solutions (Figure 2B). The higher input of vegetation was at 30 cm in amended soil whereas it was at 100 cm in reference soil solutions. The influence of amendment performed seven years before the sampling was thus still significant at 30 cm depth.

#### 2.1.2. Boron Trifluoride-Methanol Complex (BF_3_/MeOH) Derivatization: Fingerprints of Free Fatty Acids in SDOM

To identify only the free fatty acids, a derivatization with BF_3_/MeOH was then evaluated.

**Figure 3:**
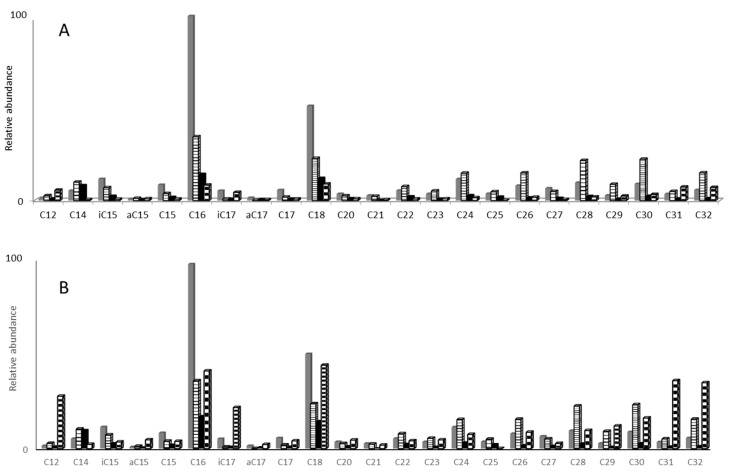
BF_3_/MeOH derivatization on 500 mL reference (**A**) and amended (**B**) soil solutions from 15 to 100 cm depth after a 40 °C evaporation step.

When performing BF_3_/MeOH derivatization on evaporated samples, the resulting distribution was only due to free fatty acids (Figure 3). The bimodal distribution ranged from C_12_ to C_32_. High concentrations in 30 cm reference soil solutions compared to thermochemolysis results showed that high concentrations of fatty acids at 100 cm were rather associated to macromolecules such as humic substances than to free molecules. This procedure is, however, not suitable for analyzing carboxylic acids of low molecular weight (<C_12_) since their increased volatility after derivatization can lead to unquantifiable losses related to evaporation.

To have a rapid overview of low molecular weight compounds, we have developed an in situ derivatization on a 2 mL sample.

### 2.2. In Situ Analysis of SDOM Fatty Acids

#### 2.2.1. Optimal In Situ Derivatization

**Figure 4:**
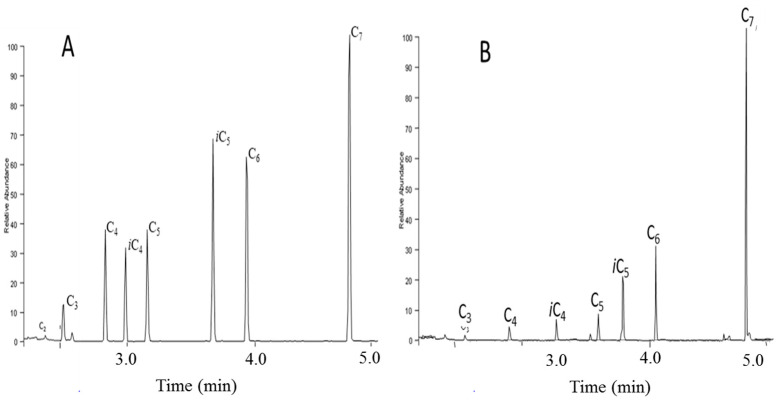
**Spectral ionic mass** (SIM) chromatogram of liquid/liquid (**A**) and direct immersion solid phase microextraction (SPME) (**B**) optimal recoveries of iso molar low molecular weight fatty acid methyl esters.

Before optimization of the derivatization step, derivatives recovery was estimated. The simple and low cost dichloromethane CH_2_Cl_2_ liquid/liquid extraction of the derivatized compounds was selected as the optimized direct immersion solid phase microextraction (DI-SPME) method that showed a peak area decreasing inversely according to the molecular weight of the compounds (Figure 4 and Figure S2).

**Figure 5:**
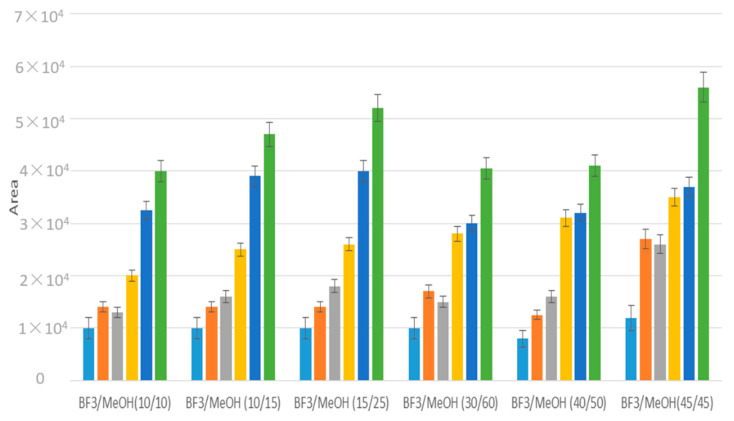
Optimization of BF_3_-MeOH and MeOH ratios (%/%) for the derivatization of a 2 mL water solution of low molecular weight fatty acids (C_4_ blue, isoC_4_ orange, C_5_ grey, iso C_5_ yellow, C_6_ dark blue, C_7_ green) with CH_2_Cl_2_ liquid/liquid extraction. Analyses were performed in triplicates.

The derivatization involving direct methylation of fatty acids with boron trifluoride (BF_3_) in methanol, used on evaporated samples, was then optimized for the direct methylation in water samples to study low molecular weight compounds. To perform in situ derivatization, different ratios of BF_3_/MeOH and MeOH (*v*/*v*) were studied (Figure 5). BF3-MeOH, MeOH 45/45 (%/%), that showed the best recoveries, was used to analyze water samples.

#### 2.2.2. SDOM Analysis by In Situ Derivatization

**Figure 6:**
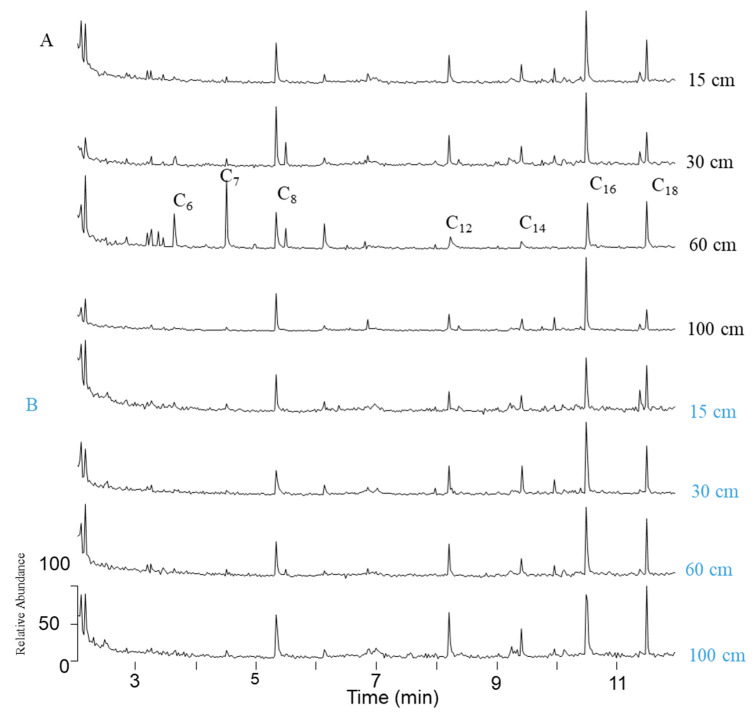
SIM chromatograms of volatile fatty acid methyl esters after BF_3_/MeOH derivatization and CH_2_Cl_2_ liquid/liquid extraction of 2 mL reference (**A**) and amended (**B**) soil solutions.

C_6_-C_18_ fatty acids were detected in all samples, when single ion monitoring analyses were performed (*m*/*z* 74, Figure 6) in 2 mL samples without any pretreament. This protocol will allow us to discriminate samples so that further analyses will be performed only on relevant samples.

## 3. Materials and Methods

### 3.1. Experimental Site

The study was conducted in the Hydrogeological Experimental Site which is a part of the Network of National Hydrogeological Sites (SNO H+) and of the French network of Critical Zone Observatories: Research and Applications (OZCAR) of the University of Poitiers located in the Regional Observatory of Deffend (OR, Mignaloux-Beauvoir, France). The field, previously managed as cereal cropping rotation (maize-wheat), is currently under grassland. The soil is a luvic cambisol fully characterized in a previous study [22]. The experimental field has been divided into 6 plots (3 reference and 3 amended, all equipped with suction cups). An amount of 150 t/ha of bio and green wastes (from the composting plant of La Villedieu du Clain, France) have been amended to follow the long-term effects of exogenous OM in the soil.

### 3.2. Percolating Water Samples and Pretreatments

The sampling was performed with 12 ceramic suction cups (31 mm, SDEC, Reignac sur Indre, France) to collect soil solutions disposed in triplicates at 5, 30, 60 or 100 cm depth. Then, the samples were filtered at 0.45 µm with a Whatman filters to separate particular to DOM. Before the storage at 4 °C for their conservation, DOC was measured. Thereafter, molecular study of dissolved fatty acids was accessible after evaporation step at 40 °C; the resulting extract was further dried in a desiccator.

### 3.3. Chemicals and Reagents

Methanol, chloroform, dichloromethane of analytical standard grade and SPME fibers were purchased from Supelco (Bellefonte, PA, USA). The fibers of carboxen/polydimethylsiloxane (CAR/PDMS), polydimethylsiloxane (PDMS), carbowax/polydimethylsiloxane divinylbenzene (CAR/PDMS/DVB) were tested. BF_3_-MeOH and tretramethylamonnium hydroxyde (TMAH) used for derivatization were purchased from Sigma-Aldrich (Darmstadt, Hessen, Germany). C_9_–C_18_ standard solution was obtained from separate compounds provided by Sigma Aldrich. Solutions were performed in methanol at 2 mM, mixed and further diluted before the experiment. Standards of low molecular weight compounds for SPME (certified reference material CRM46975) were purchased from Supelco.

### 3.4. Derivatization Process

For in situ analysis, 50 µL MeOH and 200 µL 12.5% (*w*/*v*) BF_3_-MeOH were added to 2 mL water sample and were heated during 30 min at 70 °C. Resulting fatty methyl esters (FAMES) were extracted with 200 µL CH_2_Cl_2_; 1 µL was injected for GC-MS analyses.

For dried samples, 50 μL of methanol and 200 μL of 12.5% (*w*/*v*) BF_3_-methanol were added to the vials containing the dried fatty acids (20 mg). After tightly capping the vials, they were heated for 30 min at 70 °C. Pure water (ca. 100 μL) was added and after tightly capping again, the vials were vigorously shaken and then 150 μL of dichloromethane was added. Fatty acid methyl esters were removed in the dichloromethane layer. The organic layer was transferred to a 1 mL conical vial. This procedure was repeated three times; 1 µL was injected for GC-MS analyses.

An amount of 10 mg of residues resulting from evaporation was moistened with 10 µL of a 25% (*w*/*w*) methanol solution of tetramethylamonium hydroxide (TMAH) in a cup, heated at 40 °C, 10 min and introduced in the pyrolyzer.

### 3.5. GC/MS and Pyr-GC/MS Analyses

The analyses were perform using a Thermo Finnigan Trace gas chromatograph interfaced to a Thermo Finnigan Automass mass spectrometer (Thermo Fisher Scientific, Waltham, MA, USA, 70 eV) operated in fullscan or SIM mode with a fused silica capillary column (HP-5% phenylmethylpolysiloxane 30 m × 0.25 mm, 0.25 µm film thickness) and helium as carrier gas. Line transfer and source were held, respectively, at 280 °C and 220 °C. The GC oven temperature program was from 60 °C, at 5 °C·min^−1^ up to 300 °C, and the temperature was finally held for 10 min at 300 °C. The temperatures of the injector and the detector were 250 and 300 °C, respectively.

Py-GC/MS experiments used a EGA-PY 3030D Pyrolyzer (Frontier Lab, Japan) connected to a gas chromatography instrument (GC2010 Pro, Shimadzu, Kyoto, Japan) with capillary column (SLB-5% phenylmethylsiloxan 30 m × 0.25 mm, 0.25 µm film thickness) and coupled with mass spectrometry (Ultra QP 2010). The GC and MS conditions were the same as for GC-MS analysis.

## Supplementary Materials

The following are available online, Figure S1: SPME optimal recoveries of methyl fatty esters: influence of stationary phase, time and temperature. Figure S2: (A) Mean SPME recoveries on PDMS/DVB (blue), PDMS (red) and PDMS/CAR/DVB (grey) fibers of methyl esters (10 mL, room temperature, from 5 to 60 min. (B) Histograms from room temperature to 80 °C for C_6_ (blue) and C_5_ (orange) methyl ester recoveries on PDMS/CAR/DVB fiber from 5 to 60 min exposure. * recoveries at 60 °C. (C) extraction profiles at 60 °C of C_6_ (blue) and C_5_ (orange) methyl ester on PDMS/CAR/DVB fiber from 5 to 60 min.
