# Supplementary material for: Simple Derivatization–Gas Chromatography–Mass Spectrometry for Fatty Acids Profiling in Soil Dissolved Organic Matter
Source: Molecules. 2020 Nov 12;25(22):5278. doi: 10.3390/molecules25225278 (PMC7709006; doi:10.3390/molecules25225278)
Supplement: Supplementary file 1 [file molecules-25-05278-s001.pdf]

Simple Derivatization-Gas Chromatography-Mass Spectrometry for Fatty Acids Profiling in Soil Dissolved Organic Matter

Neil Yohan Musadji[1,2] and Claude Geffroy-Rodier [2,*]

1 Université de Poitiers, Institut de Chimie des Milieux et Matériaux de Poitiers (IC2MP), UMR CNRS 7285, Equipe Eau Géochimie Santé, 4 rue Michel Brunet, 86076 Poitiers, France;

2 Institut National Supérieur d'Agronomie et de Biotechnologies (INSAB), Université des Sciences et Techniques de Masuku (USTM), 941 Franceville, Gabon

* Correspondence: claude.geffroy@univ-poitiers.fr; Tel.: +33 5 49 45 35 90

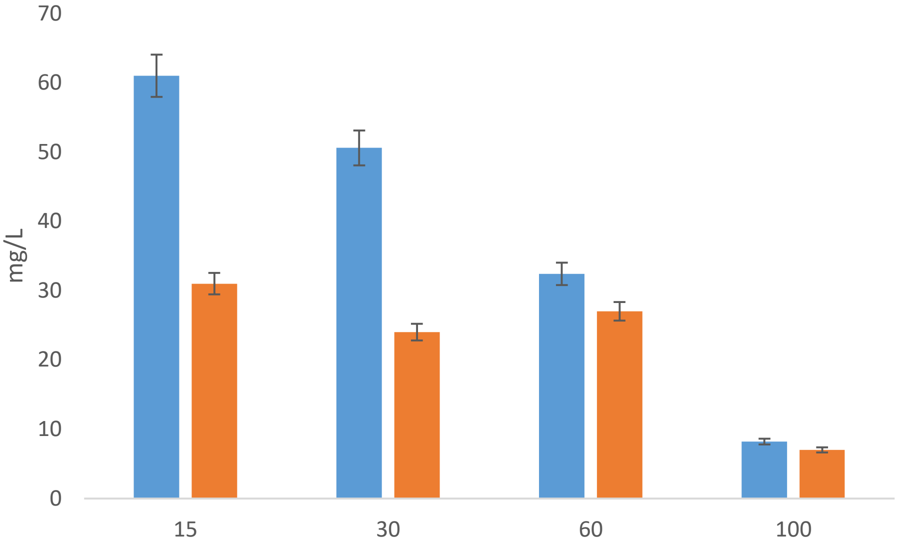

Figure S1. Mean COD values on triplicates sampled at 15, 30, 60 and 100 cm depths. For amended (blue) and reference (orange) soil solutions.

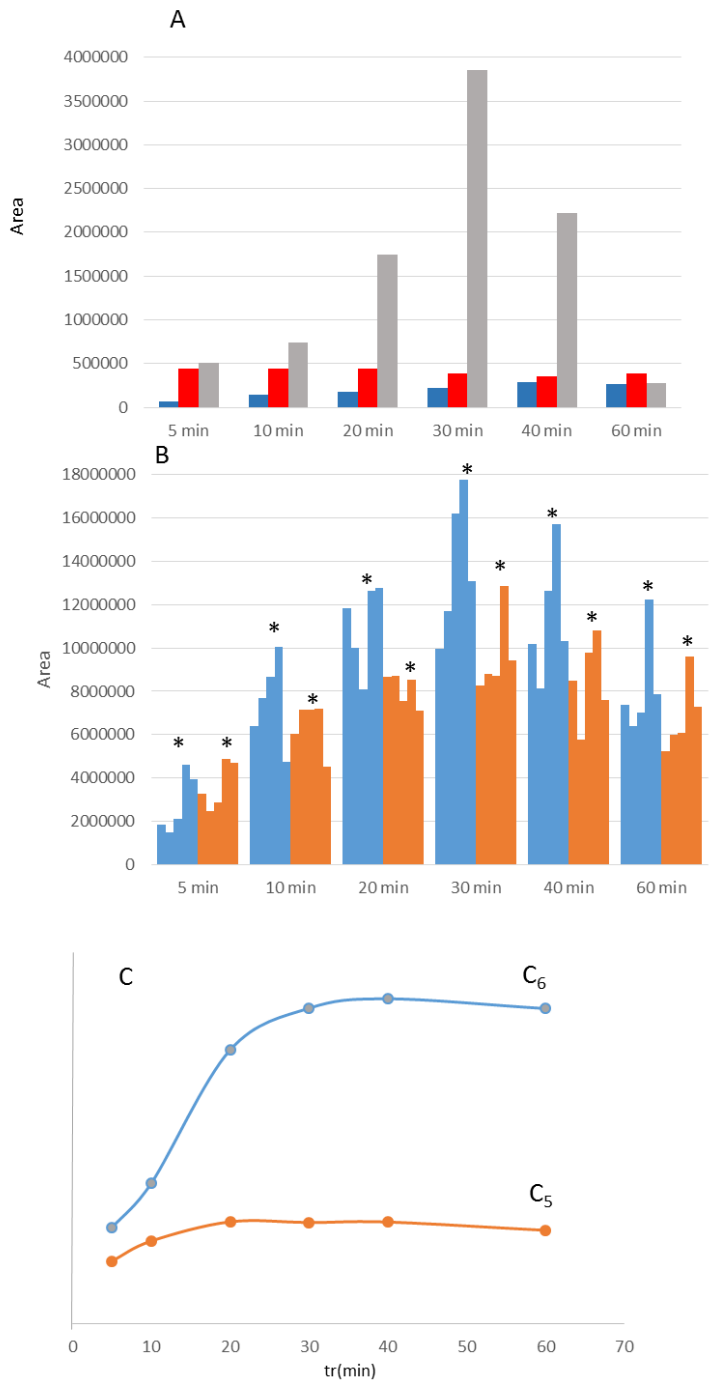

Figure S2: A) Mean SPME recoveries on PDMS/DVB (blue), PDMS (red) and PDMS/CAR/DVB (grey) fibers of methyl esters (10 mL, room temperature, from 5 to 60 min. B) Histograms from room temperature to 80°C for C_6 (blue) and C_5 (orange) methyl ester recoveries on PDMS/CAR/DVB fiber from 5 to 60 min exposure. * recoveries at 60°C. C) extraction profiles at 60°C of C_6 (blue) and C_5 (orange) methyl ester on PDMS/CAR/DVB fiber from 5 to 60 min.
